# Multiple Functions of RNA Methylation in T Cells: A Review

**DOI:** 10.3389/fimmu.2021.627455

**Published:** 2021-04-12

**Authors:** Yinong Chao, Hua-Bing Li, Jing Zhou

**Affiliations:** ^1^ Shanghai Institute of Immunology, State Key Laboratory of Oncogenes and Related Genes, Shanghai Jiao Tong University School of Medicine, Shanghai, China; ^2^ Shanghai Jiao Tong University School of Medicine - Yale Institute for Immune Metabolism, Shanghai Jiao Tong University School of Medicine, Shanghai, China

**Keywords:** RNA methylation, m^6^A, T cell, epigenetics, immune function

## Abstract

RNA modification represents one of the most ubiquitous mechanisms of epigenetic regulation and plays an essential role in modulating cell proliferation, differentiation, fate determination, and other biological activities. At present, over 170 types of RNA modification have been discovered in messenger RNA (mRNA) and noncoding RNA (ncRNA). RNA methylation, as an abundant and widely studied epigenetic modification, is crucial for regulating various physiological or pathological states, especially immune responses. Considering the biological significance of T cells as a defense against viral infection and tumor challenge, in this review, we will summarize recent findings of how RNA methylation regulates T cell homeostasis and function, discuss the open questions in this rapidly expanding field of RNA modification, and provide the theoretical basis and potential therapeutic strategies involving targeting of RNA methylation to orchestrate beneficial T cell immune responses.

## Introduction

Over 170 types of RNA modifications have been identified to date, which have allowed to expand the RNA code, giving rise to a new field called “RNA epigenetics” (1–3). Dynamic chemical modifications alter the structure and metabolism of coding and non-coding RNAs post-transcriptionally (1–3). As mass spectrometry and high throughput technology continue to be developed, these modifications can be studied more broadly and deeply. Among these modifications, RNA methylation is an important post-transcriptional regulation and comprises diverse forms of methylation, including *N* ^6^-methyladenosine (m^6^A), *N* ^6^-2’-O-dimethyladenosine (m^6^Am), *N* ^1^-methyladenosine (m^1^A), and 5-methylcytosine (m^5^C) (3). With the identification of different methyltransferase and demethylase enzymes, the physiological and pathological functions of RNA methylation have been gradually revealed, and have become an attractive area of research (4, 5).

The methylation of RNA represents one of the most ubiquitous modifications in mammalian cells and modulates multiple biological functions, especially innate and adaptive immune responses (6, 7). As crucial components of adaptive immunity, T cells are ready to prompt response to the foreign stimuli and mediate antiviral and antitumor immune responses (8, 9). Considering the importance of T cells’ immunological surveillance in the body and dynamic RNA methylation usually occurs in a very short time to initiate the regulatory role toward the target genes, it will be interesting to utilize T cell as an ideal model to understand how RNA epigenetic modifications quickly affect immune responses.

In this review, we will outline recent findings regarding the role of RNA methylation in regulating T cell function, highlight current challenges in these areas, and clarify the potential application of RNA methylation in orchestrating T cell immune responses.

## RNA Methylation in T Cells

Methylation of RNA is indispensable for the biogenesis and function of prokaryotes and eukaryotes (10). This modification is widely distributed in messenger RNA (mRNA), transfer RNA (tRNA), ribosomal RNA (rRNA), noncoding small RNA (sncRNA), and long-chain non-coding RNA (lncRNA) and can affect protein synthesis by regulating splicing, nuclear export, translation, and decay of RNA (10–13). With the discovery of m^6^A and identification of its critical role in regulating immune responses, emerging evidence has stimulated researchers to explore how other major forms of RNA methylation may modulate the host immune system, especially T cell immunity, to expand the basic understanding of the epitranscriptomic code of RNA.

### *N* ^6^-methyladenosine (m^6^A)

m^6^A refers to methylation of adenosine (A) at the nitrogen-6 position and represents one of the most frequent RNA modifications. It is preferentially enriched in 3’untranslated regions (3’UTR), long internal exons, and near stop codons of linear RNAs (14–16). The m^6^A modification is dynamically reversibly installed and removed by methyltransferases (“writers”) and demethylases (“erasers”) (17). The “writer” complex contains three core proteins: methyltransferase-like 3 (METTL3) (18, 19), METTL14 (20, 21), and Wilms’ tumor 1-associating protein (WTAP) (22, 23). Other newly recognized regulators including vir-like m^6^A methyltransferase associated (VIRMA, also called KIAA1429), RNA binding motif protein 15/15B (RBM15/15B), cbl-proto-oncogene-like protein 1 (CBLL1, also known as HAKAI), and Zinc finger CCCH-type containing 13 (ZC3H13), are essential for the nuclear localization and stabilization of the “writer” complex, or m^6^A deposition specificity (6). The two currently identified ‘‘erasers’’ are fat mass and obesity-associated protein (FTO) (24) and alkylated DNA repair protein AlkB homolog 5 (ALKBH5) (25). FTO not only functions as the demethylase for m^6^A in mRNA, but also displays demethylase activity for m^6^Am and m^1^A for specific tRNA, snRNA, or mRNA (26). Multiple proteins that bind to m^6^A, affect the fate of the corresponding RNA and its downstream functions and are referred to as m^6^A-binding proteins (‘‘readers’’). To date, three different groups of “readers” have been identified: YTH-RNA binding domain family, including three YTH-domain-family (YTHDF1-3) and two YTH domain-containing (YTHDC1-2) proteins (27–29), the heterogeneous nuclear ribonucleoprotein (HNRNP) family (30–32) and insulin-like growth factor-2 mRNA-biding proteins (IGF2BPs) (33, 34). All “readers” mediate regulatory functions of m^6^A on modified RNA and participate in RNA splicing nuclear export and storage, translation, and decay (27–29) (**Table 1**).

**Table 1:** Writers, readers and erasers in predominant RNA methylations.

RNA Modification	Writers	Erasers	Readers
m^6^A	METTL3; METTL14; WTAP; VIRMA; RBM15/15B; CBLL1; ZC3H13	FTO; ALKBH5	YTH-RNA binding domain family (YTHDF1-3, YTHDC1-2); heterogeneous nuclear ribonucleoprotein (HNRNP) family; insulin-like growth factor-2 mRNA-biding proteins (IGF2BPs)
m^6^Am	PCIF1	FTO	Not defined
m^1^A	TRMT6/TRMT61A; TRMT61B; TRMT10C; NML	FTO; ALKBH1; ALKBH3	YTHDF1-3; YTHDC1
m^5^C	NSUN1-7; DNMT2	Not defined	ALYREF; YBX1

T cells, generally comprising CD4^+^ T and CD8^+^ T cells, largely represent the foundation of the adaptive immune system. Naïve CD4^+^ T cells exhibit features of stem cells, and will further differentiate into different T helper (Th) cell subsets upon stimulation by various microenvironmental signals (35, 36). Evidence from embryonic stem cells has indicated the importance of m^6^A in determining stem cell fates (37), implying the potential of m^6^A in directing Th cell differentiation. In 2017, our lab constructed a conditional knockout mouse model (*Mettl3* ^flox/flox^ *Cd4* ^Cre^) for m^6^A writer protein-METTL3 in T cells, in the first attempt to elucidate the *in vivo* role of METTL3 and consequently the m^6^A RNA modification in regulating mouse T cells homeostasis and differentiation (38). Characterization of immune cell populations at steady state of this mice model revealed that the ablation of METTL3 in T cells disrupted T cell homeostasis (38). Furthermore, METTL3-deficient naïve CD4^+^ T cells differentiated into fewer Th1 and Th17 cells, more Th2 cells, without affecting regulatory T cells (Tregs) induction compared with WT naïve CD4^+^ T cells (38). Interestingly, METTL3 or METTL14 also promoted naïve CD4^+^ T cell homeostatic expansion *in vivo* during the adoptive transfer colitis model (38). With these observations, we determined that the mRNAs of the suppressor of cytokine signaling (SOCS) family genes modified by m^6^A underwent rapid mRNA degradation upon interleukin (IL)-7 stimulation, thus initiating the homeostasis and differentiation of naïve T cells by relieving the block on the IL-7-signal transducers and activators of transcription 5 (STAT5) pathway (38). Shortly after, our omics data was re-evaluated by others, who comprehensively quantified the RNA dynamics of T cell during differentiation, and showed that m^6^A depletion impairs this process (39), further illustrating the importance of the epitranscriptomic m^6^A modification, in governing T cell homeostasis and function.

Although *in vitro* evidence suggests the dispensable role of METTL3 in directing Tregs differentiation by a T cell receptor (TCR)-dependent T cell induction system (38), whether m^6^A modification affects Tregs functions *in vivo* is still unclear. After observing that *Mettl3* ^flox/flox^ *Cd4* ^Cre^ mice developed spontaneous chronic inflammation in the intestine after 3 months of age, we hypothesized that Tregs might have impaired repressive functions in these mice (40). Therefore, we bred *Mettl3* ^flox/flox^ *Foxp3* ^Cre^ mice to specifically delete METTL3 expression in Tregs, and observed the development of severe systemic autoimmune diseases of the mice soon after weaning. The mice began to die at the ages of 8-9 weeks old, which was attributed to the systematic loss of Tregs suppressive function lacking m^6^A modification (40). Mechanistically, the METTL3-mediated m^6^A RNA modification specifically targets the SOCS gene family and sustains the suppressive functions of Tregs *via* the IL2-STAT5 pathway (40). The findings further implied that T cell-specific delivery of m^6^A-modifying agents might be employed in treating autoimmune disorders.

T follicular helper T (Tfh) cells, as a specialized CD4^+^ T cell subset, initiate germinal center (GC) formation and promote humoral immunity (41), whether their lineage differentiation can be directed by m^6^A modification remains to be identified. One study indicated that the knockdown of METTL3 or METTL14 in CD4^+^ T cells with short hairpin RNA (shRNA) could promote Tfh development upon lymphocytic choriomeningitis virus (LCMV) infection (42). However, other studies reported an opposite phenotype and pointed out that conditional ablation of METTL3 in CD4^+^ T cells dampened Tfh differentiation, proliferation, and survival ability after LCMV challenge (43). Notably, the latter study indicated that METTL3-mediated m^6^A modification of transcription factor 7 (TCF7) mRNA can stabilize the transcript, and therefore maintain protein expression of TCF7 to initiate and secure the differentiation of Tfh cells (43). This regulatory mechanism differs with naïve CD4^+^ T cells or Tregs, where METTL3-directed m^6^A modification decreases mRNA stabilization (38, 40). The divergent functional outcome of m^6^A modification mainly depends on diverse m^6^A “readers” in a cell type and cellular context-dependent fashion. Therefore, a deeper evaluation of the phenotypic effects of different m^6^A-readers will advance our knowledge of the m^6^A machinery.

m^6^A methylation may modulate T cells either directly or indirectly by affecting the function of antigen-presenting cells (APCs) (44, 45). In YTHDF1-deficient mice, the antigen-specific CD8^+^ T cell antitumor response increased compared with wild-type mice, relying on enhanced cross-presentation of tumor antigens and cross-priming of CD8^+^ T cells by classical dendritic cells (cDCs) (44). Furthermore, METTL3-specific deficiency in DCs led to phenotypic and functional maturation defects, causing a reduction in levels of co-stimulatory molecules CD40, CD80, and cytokine IL-12, and reduced the ability to stimulate CD4^+^ T cell responses (45). These findings raise the possibility of exploiting m^6^A methylation to promote DC activation and DC-based T cell responses. However, whether the m^6^A machinery directly regulates the development and function of CD8^+^ T cells has yet to be determined.

**Figure 1:**
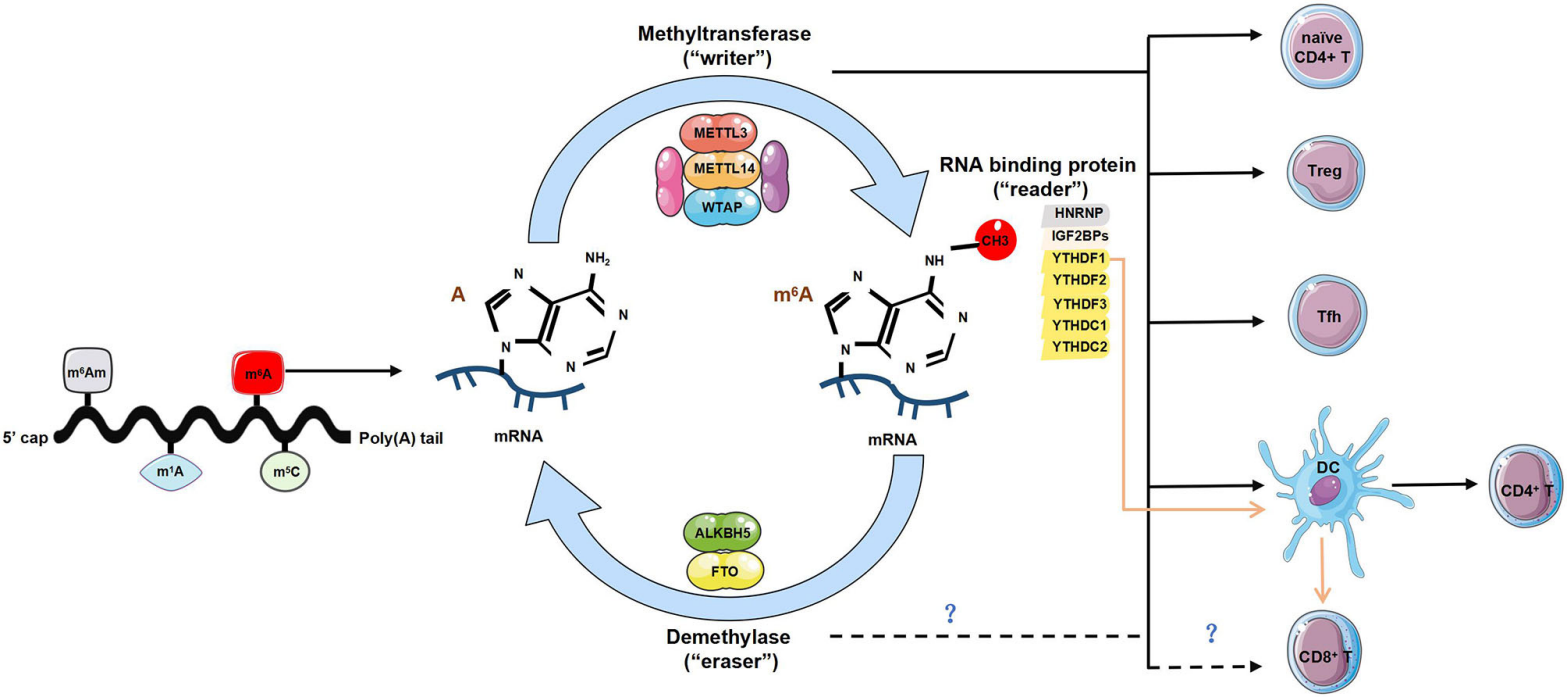
The regulatory role of m^6^A modification in T cells. Diverse forms of RNA methylation have been identified on mRNAs, mainly involving *N* ^6^-methyladenosine (m^6^A), *N* ^6^-2’-O-dimethyladenosine (m^6^Am), *N* ^1^-methyladenosine (m^1^A) and 5-methylcytosine (m^5^C). m^6^A modification is reversibly installed and removed by methyltransferases (“writers”) and demethylases (“erasers”). Multiple proteins that bind to m^6^A to affect the fate of RNA are referred to as m^6^A-binding proteins (‘‘readers’’). The “writers” complex is mainly composed of METTL13, METTL14 and WTAP, can directly modulate T cells by maintaining homeostasis of naïve CD4^+^ T cells, promoting the function of regulatory T cells (Tregs) and T follicular helper (Tfh) cells. It also indirectly enhances CD4^+^ T cells function via promoting dendritic cell (DCs) activation and DC-based T cell response. The “readers” involve YTHDF1-3, YTHDC1-2, HNRNP and IGF2BPs. The lack of YTHDF1 can enhance antigen-specific CD8^+^ T cell antitumor response indirectly by the increased cross-priming of CD8^+^ T cells by DCs. However, whether the “writers” and “readers” directly regulates the development and function of CD8^+^ T cells is unknown. The current identified two ‘‘erasers’’ are FTO and ALKBH5, whether and how “erasers” control T cell homeostasis or functionality is yet to be determined.

Although current studies have highlighted the importance of m^6^A in governing T cell functions, it seems that these have only focused on better defining the role of the “writers” or “readers” (**Figure 1**). It will be intriguing to characterize whether and how “erasers” control T cell homeostasis or functionality. It has been reported that dopamine receptors expressed in T cells are critical for instructing thymic T cell development and maintaining T cell function (46, 47). Interestingly, FTO can also participate in the dopamine signaling pathway (48); thus, it remains a possibility that FTO may affect T cell homeostasis or function *via* dopamine receptors. Recent studies have excluded the potential role of ALKBH5 in directing Tfh differentiation upon LCMV infection using shRNA-mediated knockdown of ALKBH5 (42). However, based on results obtained from macrophages, it has been suggested that ALKBH5 demethylates α-ketoglutarate dehydrogenase (OGDH) transcript, increases its mRNA stability and protein expression, and metabolically promotes viral replication in macrophage (49), which still raises the possibility of ALKBH5 in maintaining T cell function and warrants additional investigation.

### *N* ^6^-2’-O-dimethyladenosine (m^6^Am)

m^6^Am refers to the terminal modification of the first nucleotide after the mRNA 5’cap, and was first identified in animal cells and viral mRNA in 1975 (50). Usually, if this first nucleotide is 2-O-methyladenosine (Am), it can be further methylated at the nitrogen-6 position to form m^6^Am (50). Like m^6^A, m^6^Am is also dynamically regulated by methyltransferase and demethylase (51, 52). Phosphorylated CTD interacting factor 1 (PCIF1) is the only evolutionarily conserved methyltransferase for m^6^Am, and depleting PCIF1 leads to the deficiency of m^6^Am alone without affecting m^6^A levels or distribution (52–54). The function of PCIF1 is still controversial as one study indicated the loss of PCIF1 did not affect mRNA translation but reduced stability of a subset of m^6^Am-annotated mRNAs (52), while another reported that PCIF1 suppressed protein translation without influencing mRNA stability (55). The inconsistency may result from the different experimental systems used to dissect the effects of m^6^Am on translation, thus, it remains to be determined whether this effect is maintained, diminished, or enhanced under various biological circumstances.

Contrary to the specificity of ALKBH5 toward m^6^A modification, FTO shows wide reversible demethylation activity, and functions as a demethylase for the m^6^Am modification to reduce the stability of target mRNAs, preferentially demethylating m^6^Am rather than m^6^A (26, 56) (**Table 1**). Considering the possible role of FTO in maintaining T cell development or function (48), additional studies are needed to characterize m^6^Am’s function in orchestrating T cell immunity.

### *N* ^1^-methyladenosine (m^1^A)

The methylation on the nitrogen-1 position of adenosine to form m^1^A was first identified in tRNA (57). This modification is typically found at position 58 in the T-loop of tRNA (m^1^A58) (58, 59). The m^1^A modification on tRNA is of great importance for tRNA folding, stability, and tRNA-protein interaction (60–62). m^1^A has also been detected in rRNA and affects the tertiary structure of ribosomes and the translation of downstream genes (63, 64). Subsequently, the rare presence of m^1^A sites in mRNA and lncRNA was also reported (65, 66). Recent findings suggest that m^1^A at the coding sequence (CDS) in mitochondrial messenger RNA (mt-mRNA) blocks the effective translation of modified codons (66, 67). Moreover, ribosome profiling data indicate that m^1^A in nuclear mRNA might promote translation (67). However, the exact function of m^1^A in these RNAs still need further investigation.

m^1^A, as a reversible RNA modification, is catalyzed by methyltransferases including tRNA methyltransferase 6/61A (TRMT6/61A), TRMT61B, TRMT10C, and Nucleomethylin (NML), and by demethylases FTO, ALKBH1, and ALKBH3 (26, 58, 59, 67–70). YTHDF1-3 and YTHDC1 have been reported as the “readers” of m^1^A (71) (**Table 1**). TRMT6/61A is responsible for the modification of a series of m^1^A sites in tRNA and lncRNA, recognizing the substrate RNA mainly through a strong T-loop structure typically comprising a 5-base pair (bp) stem and a 7-bp loop (67, 72). TRMT61B, as a mitochondrial-specific tRNA methyltransferase, has been reported to catalyze m^1^A modification in both tRNA and rRNAs (73, 74). TRMT10C can catalyze m^1^A modification in mitochondrial coding transcripts (75, 76), and NML will catalyze the formation of m^1^A at multiple rRNA sites both in human and mouse cells (77). Although distinct tRNA expression patterns and dynamic changes involving tRNA modification occur in early mouse CD4^+^ T cells activation, the m^1^A modification at position 58 of tRNA remains constant throughout this process (78), questioning whether m^1^A may regulate T cell activation. Therefore, uncovering the biological consequences of m^1^A under various physiological and pathological conditions in T cells will be critical to improve our understanding of the role that m^1^A machinery plays in regulating T cell immunity.

### 5-Methylcytosine (m^5^C)

The existence of m^5^C in RNAs has been known since the 1970s (79, 80). High throughput detection methods identified m^5^C as an abundant RNA modification in diverse RNA species, including mRNA, tRNA, rRNA, and other ncRNAs (81). Like m^6^A, the m^5^C modification in RNA is also catalyzed by “writers”, including DNA methyltransferase homologs and members of the NOL1/NOP2/SUN domain (NSUN) family proteins (including NSUN1-7) and DNA methyltransferase (DNMT) homologue DNMT2 (81–84), and “readers” like Aly/REF export factor (ALYREF) (85) and Y-box binding protein 1(YBX1) (86). However, the “erasers” of m^5^C in RNAs have not yet been identified (**Table 1**). m^5^C has emerged as a critical regulator involved in modulating the export and stability of RNA, ribosome assembly, and translation (81, 87). However, the specific gene signatures of m^5^C-related regulators in T cell immunity remain largely unknown.

A recent study has reported reduced mRNA m^5^C levels in CD4^+^ T cells from patients with systemic lupus erythematosus (SLE) compared with CD4^+^ T cells of healthy controls (HCs) (88). NSUN2 is the only known mRNA m^5^C methyltransferase (75) and whose mRNA and protein expression is reduced dramatically in CD4^+^ T cells from SLE patients relative to HCs (88). Importantly, m^5^C hypomethylated transcripts in SLE stable groups or SLE moderate/major active groups have shown obvious enrichment of eukaryotic translation elongation and protein methylation (88). In addition, up-regulated genes presenting an abundance of m^5^C has been described to be involved in the flares and remission of SLE patients, and subsequent damage to the patient’s immune system (88). This study suggested the relevance of aberrant m^5^C mRNA modification in vital immune pathways of CD4^+^ T cells from SLE patients, but how the “writer” NSUN2 contributes to the m^5^C epitranscriptomic code during SLE is still unclear. Further studies are needed to acquire a better understanding of the role of NSUN2 in patients with SLE. Notably, other RNA methylations, such as 3-methylcytidine (m^3^C), *N* ^1^-methylguanosine (m^1^G), 5-methyluridine (m^5^U) have also been mapped in CD4^+^ T cells (88), and it would be interesting to explore whether these modifications exert a similar regulatory role with m^6^A in maintaining T cell homeostasis in the future.

## Concluding Remarks

As a critical component of epigenetics, posttranscriptional RNA methylation provides abundant possibilities for different physiological and pathological processes relevant to T cells. Based on the complex self-regulatory processes in T cells, major knowledge gaps remain to be filled in this developing field. Studies in mice have deciphered the significance of m^6^A methylation as a “brake” to modulate transcription during T cell activation (38). Although many m^6^A sites have been detected on T cells, whether these sites are subject to an equal contribution from the action of “writers” and “erasers” needs to be further determined. Further, considering the importance of ‘‘writers’’ in maintaining T cell function, the role of m^6^A ‘‘erasers’’ in governing T cell homeostasis and function remains to be elucidated. Future studies are required to answer whether a selectivity and asymmetry in the actions of m^6^A “writers” and “erasers” is active in controlling T cell function.

Since our observations regarding the cross-talk between RNA modifications in colorectal cancer highlighted their therapeutic liability toward immunotherapy (89), it would be interesting to decipher the existence of a similar complex regulatory network in T cells, and to determine whether other RNA methylations might function as a sort of “gas pedal” or “pace keeper” to control T cell clonal expansion, differentiation, and subsequent effector functions. The identification of a specific RNA epigenetic translational checkpoint will advance our knowledge concerning how different RNA methylations are sequentially coordinated to regulate T cell immunity. Moreover, a proper investigation with the objective to explain why and under which conditions T cells rely on different types of RNA methylations to regulate gene expression will be a major step forward. Future explorations will reveal novel therapeutic targets by exploiting RNA methylations to alleviate T cell-related inflammatory diseases, infections, and to promote cancer immunotherapy.

## Author Contributions

YC drafted the manuscript. HBL and JZ designed the review, wrote, and revised the manuscript. All authors contributed to the article and approved the submitted version.

## Funding

This work was supported by the National Natural Science Foundation of China (91753141/82030042/32070917 to HBL, 81901569 to JZ), Shanghai Science and Technology Committee (20JC1417400/201409005500/20JC1410100 to HBL), the Postdoctoral Innovation Talent Support Program (BX20190214 to JZ), the Shanghai Super Postdoctoral Program (JZ), China Postdoctoral Science Foundation (2020M671149 to JZ), the Program for Professor of Special Appointment (Eastern Scholar) at Shanghai Institutions of Higher Learning (HBL), the start-up fund from the Shanghai Jiao Tong University School of Medicine (HBL).

## Conflict of Interest

The authors declare that the research was conducted in the absence of any commercial or financial relationships that could be construed as a potential conflict of interest.
